# ECL 3.0: a sensitive peptide identification tool for cross-linking mass spectrometry data analysis

**DOI:** 10.1186/s12859-023-05473-z

**Published:** 2023-09-20

**Authors:** Chen Zhou, Shuaijian Dai, Shengzhi Lai, Yuanqiao Lin, Xuechen Zhang, Ning Li, Weichuan Yu

**Affiliations:** 1 Department of Electronic and Computer Engineering, The Hong Kong University of Science and Technology, Clear Water Bay, Hong Kong, China; 2 Division of Life Science, The Hong Kong University of Science and Technology, Clear Water Bay, Hong Kong, China; 3 HKUST Shenzhen-Hong Kong Collaborative Innovation Research Institute, Shenzhen, China

**Keywords:** Proteomics, Cross-linking mass spectrometry, Database searching, Protein feedback

## Abstract

**Background:**

Cross-linking mass spectrometry (XL-MS) is a powerful technique for detecting protein–protein interactions (PPIs) and modeling protein structures in a high-throughput manner. In XL-MS experiments, proteins are cross-linked by a chemical reagent (namely cross-linker), fragmented, and then fed into a tandem mass spectrum (MS/MS). Cross-linkers are either cleavable or non-cleavable, and each type requires distinct data analysis tools. However, both types of cross-linkers suffer from imbalanced fragmentation efficiency, resulting in a large number of unidentifiable spectra that hinder the discovery of PPIs and protein conformations. To address this challenge, researchers have sought to improve the sensitivity of XL-MS through invention of novel cross-linking reagents, optimization of sample preparation protocols, and development of data analysis algorithms. One promising approach to developing new data analysis methods is to apply a protein feedback mechanism in the analysis. It has significantly improved the sensitivity of analysis methods in the cleavable cross-linking data. The application of the protein feedback mechanism to the analysis of non-cleavable cross-linking data is expected to have an even greater impact because the majority of XL-MS experiments currently employs non-cleavable cross-linkers.

**Results:**

In this study, we applied the protein feedback mechanism to the analysis of both non-cleavable and cleavable cross-linking data and observed a substantial improvement in cross-link spectrum matches (CSMs) compared to conventional methods. Furthermore, we developed a new software program, ECL 3.0, that integrates two algorithms and includes a user-friendly graphical interface to facilitate wider applications of this new program.

**Conclusions:**

ECL 3.0 source code is available at https://github.com/yuweichuan/ECL-PF.git. A quick tutorial is available at https://youtu.be/PpZgbi8V2xI.

**Supplementary Information:**

The online version contains supplementary material available at 10.1186/s12859-023-05473-z.

## Background

Cross-linking mass spectrometry (XL-MS) is an emerging technology in the field of proteomics that provides insights into protein–protein interactions (PPIs) and protein structures. The significance of XL-MS lies in its ability to detect PPIs that are weak, transient, or difficult to study using other methods such as co-immunoprecipitation or yeast two-hybrid assays. Compared to X-ray crystallography or nuclear magnetic resonance (NMR) spectroscopy, XL-MS needs less complex sample preparation and provides much higher throughput in the structural study [1].

However, one of the major challenges of XL-MS is the low peptide fragmentation efficiency in the collision-induced dissociation (CID) process. During the process, longer (or heavier) cross-linked peptides often impede the fragmentation of shorter (or lighter) cross-linked peptides, resulting in less informative fragment ions in the MS/MS spectrum [2, 3]. To address this problem, researchers have proposed to design novel cross-linkers, refine experimental procedures and optimize analysis algorithms.

We proposed a protein feedback idea in the cleavable cross-linking data analysis and used the protein-peptide association to help identify insufficient fragmented peptides [4]. This method has improved the sensitivity in the cleavable cross-linking data.

The implementation of the protein feedback method to the non-cleavable cross-linking data can make more impact in the field because the majority of XL-MS experiments still uses non-cleavable cross-linkers [5, 6]. In this paper, we implement the protein feedback idea in the analysis of non-cleavable cross-linking MS data based on our previously developed non-cleavable data analysis tools [7–9]. We further integrate both non-cleavable and cleavable tools into a unified software program with a user-friendly graphic interface. We call the new program ECL 3.0. Experimental results have demonstrated the superior performance of ECL 3.0 over existing standard analysis tools.

**Fig. 1:**
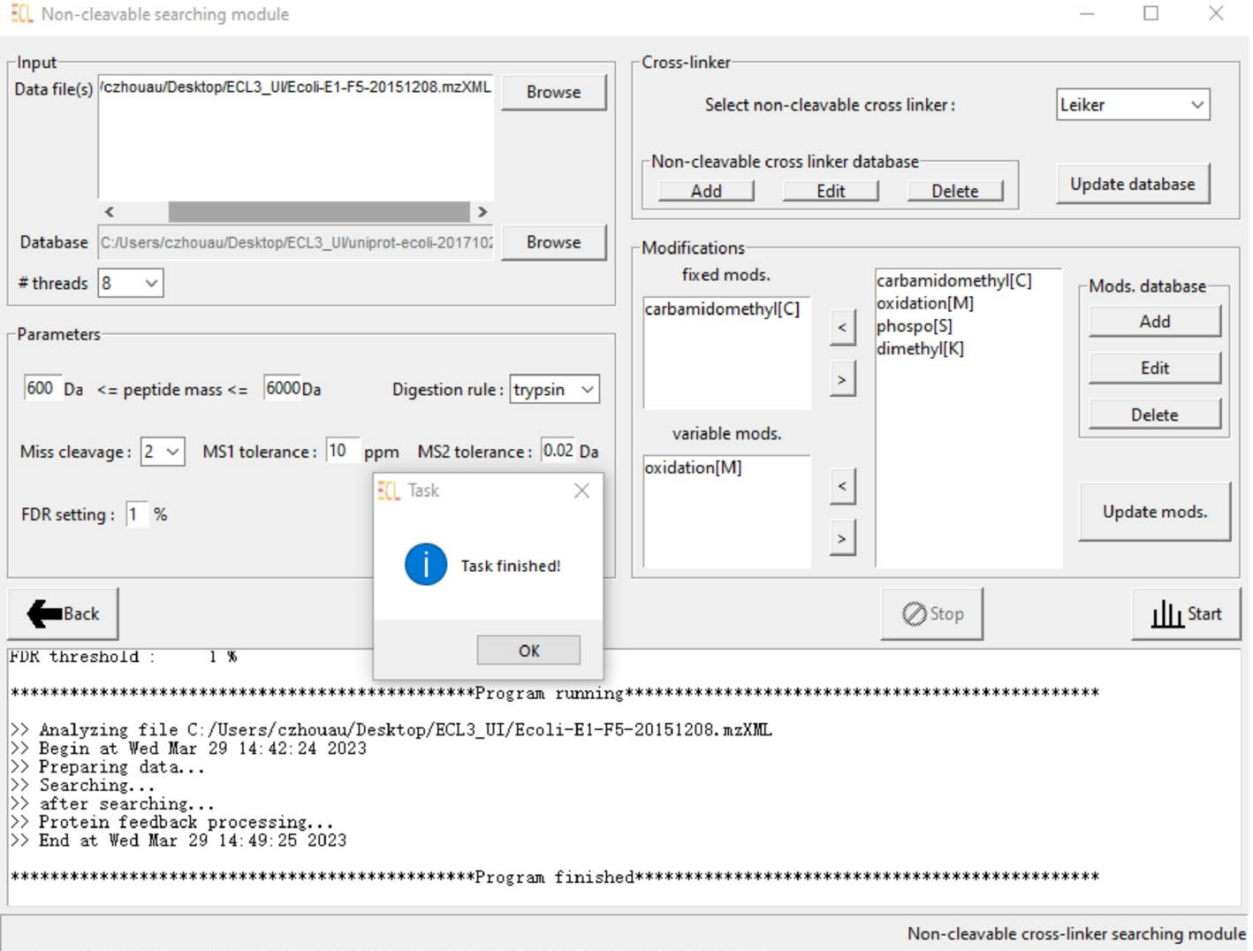
A snapshot of graphical user interface (GUI) in ECL 3.0

## Implementation

In XL-MS, imbalanced fragmentation of cross-linked peptides leads to low-quality XL-MS spectra. Although designing suitable scoring functions can help identify correct peptides, scoring function does not completely solve the problem, especially when no fragmented ions of the shorter or lighter peptide are available. To address this issue, we propose to use additional (global) information in peptide identification.

Suppose two similar peptides are ranked as the top hits by a scoring function with no preference for one over the other. One peptide has many sibling peptides identified from other spectra, whereas the other does not have any sibling peptides being identified elsewhere. Because sibling peptides from the same protein should have higher chances to appear in other MS/MS spectra than some irrelevant peptides, we propose to add more weight to a peptide with many sibling peptides than a peptide without sibling peptides. We define this adjustment as the protein feedback method [4]. For completeness, we briefly summarize the implementations of the protein feedback method below. The reader is referred to [4] for mathematical details:

1. In XL-MS, each query spectrum undergoes preliminary identification of potential peptides using a scoring function. The top *N* (default 20) cross-linked candidate pairs are then cached for examination. Subsequently, we apply a filtering step to retain only the highest-scored candidate and those whose score is at least 80% of the top one.
2. The first hit in each spectrum is collected and filtered based on a score cutoff, which is determined empirically and varies depending on the specific scoring function used.
3. The filtered peptides are considered the “true” positives temporarily and are used to build a protein score database. Each of these “true” positives contributes weight to its corresponding protein score [4].
4. After building the protein score database, the top *N* pairs of each spectrum are re-ranked by their protein scores. And the cross-linked pair with the highest protein score is considered the correct cross-link spectrum match (CSM).
5. After the adjustment of protein feedback in the peptide-spectrum matching process, the target-decoy approach is used to control the false discovery rate (FDR) [10].

ECL 3.0 is an extension of ECL-PF [4], which was focused exclusively on cleavable cross-linking search. In addition to ECL-PF, ECL 3.0 includes Xolik [9], a non-cleavable searching program we previously designed, and incorporates protein feedback. The main code modification in the original ECL-PF cleavable module was the output format. Both ECL-PF and Xolik are standardized to ensure consistent output results.

ECL 3.0 is written in conjunction with Python3 and C++. We provide a testing dataset at http://bioinformatics.hkust.edu.hk to help users get started with the software. Additionally, we have created a detailed tutorial with step-by-step instructions for using ECL 3.0, which can be accessed at youtu.be/PpZgbi8V2xI.

## Results and discussion

**Fig. 2:**
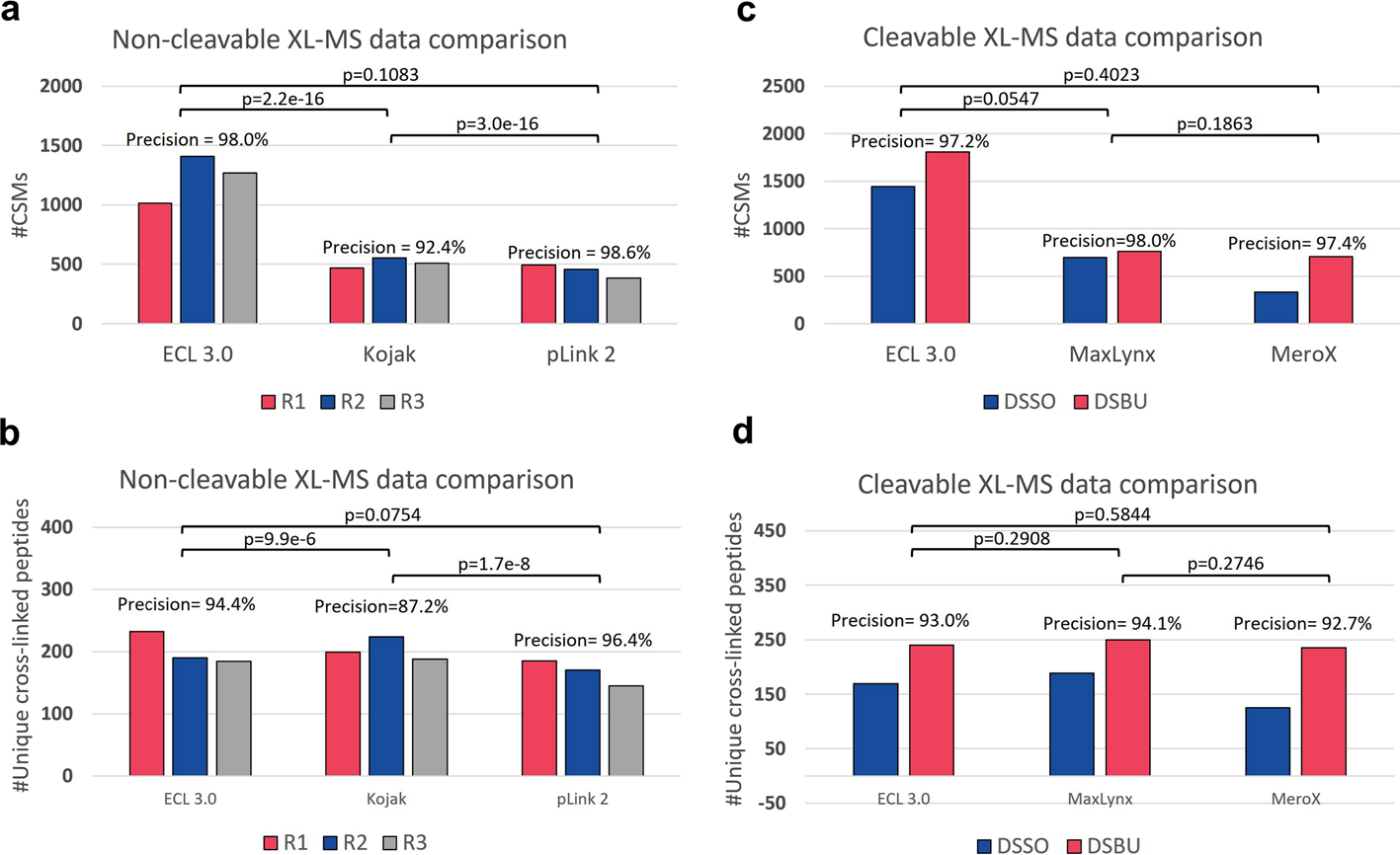
Performance comparison results. **a**, **b** The performance of ECL 3.0, Kojak, and pLink 2 in non-cleavable data analysis using three technical replicates (R1, R2, and R3) of a synthetic peptide sample (PXD014337) [15], which uses disuccinimidyl suberate (DSS) as the cross-linker. The number of cross-linked peptide spectrum matches (CSMs), the number of unique cross-linked peptides and precision (TP/TP+FP) were calculated for each software tool. ECL 3.0 identified over twice as many results as other tools in the CSMs number and similar results in the unique cross-linked peptides. Using the (one-sided) Fisher’s exact test, the *p*-value indicated that ECL 3.0 has significantly better precision than Kojak and has similar precision as pLink 2. **c**, **d** Identification results of ECL 3.0, MaxLynx, and MeroX using cleavable data. Results using two different cross-linkers (disuccinimidyl sulfoxide (DSSO) and disuccinimidyl dibutyric urea (DSBU)) are shown. ECL 3.0 identified over twice as many results as other tools in CSMs numbers and similar results in the unique cross-linked peptides. The precision of these three tools does not have a significant difference according to the (one-sided) Fisher’s exact test (*p*-value)

Figure 1 illustrates the graphical user interface (GUI) of ECL 3.0. Figure 2 shows comparison results between ECL 3.0 and four other state-of-the-art methods. In non-cleavable XL-MS data analysis, ECL 3.0 was compared with Kojak [11] and pLink 2 [12]. In cleavable XL-MS data analysis, ECL 3.0 was compared with MaxLynx [13] and MeroX [14]. The comparison was performed using a synthetic data set [15], and the results from cleavable data analysis were redrawn from [4]. ECL 3.0 achieves significantly higher sensitivity in terms of the number of CSMs with a similar level of precision. For the unique cross-linked peptides, all of the tools have similar levels of sensitivity. This is due to the fact that this data set is of high quality and only contains hundreds of synthesized peptides. We further tested these tools on larger and more complicated real datasets of human proteins. The result (Additional file 1: Fig. S5) reveals that ECL 3.0 can identify 49% more unique cross-linked peptides than other software.

We have done three different types of validation using real data sets in [4], including using the Protein Data Bank (PDB) structure to verify the result, using the protein–protein interactions (PPIs) database (BioGRID and DroID) to verify the protein interactions, and using the artificially created junk data set as negative control to check the false positives. During the revision of this manuscript, we are inspired by the reviewer’s suggestions and further use another way to validate the result of ECL 3.0 (in Additional file 1). These validations depict that ECL 3.0 improves the sensitivity of cross-linking peptide identification without sacrificing precision.

Details of the parameter settings and further experimental comparisons can be found in the Additional file 1, where we further utilized two E. coli data sets [12, 16] and four human data sets [17–20].

## Conclusion

ECL 3.0 is a comprehensive cross-linking mass spectrometry data analysis tool. It enables us to unravel more intriguing PPIs and protein structure conformations by using the protein feedback mechanism. ECL 3.0 is available with GUI version and is open source at https://github.com/yuweichuan/ECL-PF.git.

## Availability and requirements

Project name: ECL 3.0

Project home page: https://github.com/yuweichuan/ECL-PF.git

Project alternative page: https://zenodo.org/record/8176558

Operating system(s): Windows.

Programming languages: Python and C++.

Other requirements: Python 3.6 or higher.

License: MIT

Any restrictions to use by non-academics: None

### Supplementary Information

**Additional file 1.** Further comparisons, validations, and supplementary figures.

## Data Availability

All the data sets used in the comparison can be found on the public proteomic database (PRIDE Archive) at https://www.ebi.ac.uk/pride/archive/. The source code is available at https://github.com/yuweichuan/ECL-PF.git.

## Abbreviations

XL-MS: Cross-linking mass spectrometry
PPIs: Protein–protein interactions
MS/MS: Tandem mass spectrum
CSMs: Cross-link spectrum matches
NMR: Nuclear magnetic resonance
CID: Collision-induced dissociation
FDR: False discovery rate
GUI: Graphical user interface
DSS: Disuccinimidyl suberate
DSSO: Disuccinimidyl sulfoxide
DSBU: Disuccinimidyl dibutyric urea
