# Supplementary material for: ECL 3.0: a sensitive peptide identification tool for cross-linking mass spectrometry data analysis
Source: BMC Bioinformatics. 2023 Sep 20;24:351. doi: 10.1186/s12859-023-05473-z (PMC10510197; doi:10.1186/s12859-023-05473-z)
Supplement: Supplementary file 1 — Additional file 1. Further comparisons, validations, and supplementary figures. [file 12859_2023_5473_MOESM1_ESM.pdf]

SUPPORTING INFORMATION

ECL 3.0: a sensitive peptide identification tool for cross-linking mass spectrometry data analysis

Chen Zhou[1], Shuaijian Dai[1], Shengzhi Lai[1], Yuanqiao Lin[1], Xuechen Zhang[1], Ning Li[2,3] and Weichuan Yu[1,3,⋆]

[1] Department of Electronic and Computer Engineering,
The Hong Kong University of Science and Technology, Hong Kong, China

[2] Division of Life Science,
The Hong Kong University of Science and Technology, Hong Kong, China

[3] HKUST Shenzhen-Hong Kong Collaborative Innovation Research Institute,
Futian, Shenzhen, China

[⋆] Correspondence: W.Y. (eeyu@ust.hk)

Contents

Contents **2**

Table S1. Parameters used in the analysis of non-cleavable data set (PXD014337). . . 5

Table S2. Parameters used in the analysis of E. coli data sets. 6

Figure S1. Performance using E. coli data sets. 7

Figure S2. Venn diagrams of E. coli data sets on CSMs number. 8

Figure S3. Investigation of difference between non-overlapping and overlapping CSMs. . 9

Table S3. Parameters used in the analysis of human data sets. 11

Figure S4. CSMs and Venn diagrams for human data sets. 12

Figure S5. Number of Unique cross-linked peptides and Venn diagrams for human data sets. 13

E. coli data set comparison

The parameter setting for the non-cleavable data comparison in the main file is shown in Table. S1. Our previous paper [1] demonstrated the validity of ECL 3.0 in the cleavable data analysis module. In this supporting file, we provide a comparison of ECL 3.0's non-cleavable searching module using the E. coli dataset from [2]. We compared ECL 3.0, Kojak [3], and pLink 2 [2] using the parameters shown in Table. S2. Fig. S1 shows that ECL 3.0 identified the highest number of cross-linked peptide spectrum matches (CSMs) in each individual data set. Additionally, Venn diagrams in Fig. S2 reveal that ECL 3.0 covers 73% of the results from the other tools in addition to its own unique results.

Investigation of uniquely identified non-overlapping CSMs

The comparison of the E. coli data set (Venn diagrams in Fig. S2) demonstrated that ECL 3.0 can identify a substantial number of unique cross-linked spectrum matches (CSMs), accounting for nearly 40% of the union results obtained from ECL 3.0, Kojak, and pLink 2. To investigate these unique spectra further, we analyzed their characteristics.

Initially, we treated the union cross-link results of Kojak and pLink 2 as our reference set1. We found that 90.8% of the non-overlapping CSMs in ECL 3.0 have corresponding cross-links in this reference set1, indicating that ECL 3.0 identified a considerable number of redundant cross-links. Additionally, we extracted all the results from Kojak without applying false discovery rate (FDR) control, creating a non-quality control reference set2. Within the non-overlapping results of ECL 3.0, we observed that 3.5% share the exact same cross-linked peptides as those in reference set2, albeit with FDRs exceeding 0.01. Furthermore, the rest 5.7% of the non-overlapping CSMs in ECL 3.0 exhibited one cross-linked peptide present in the reference set2, while the other one did not match. None of the CSMs in ECL 3.0 showed both cross-linked peptides not mapping to the reference set2.

To further comprehend why these non-overlapping spectra remain undetected by other tools, we compared the scores of overlapping CSMs with those of non-overlapping CSMs. The score histogram depicted in Fig.S3(a) reveals that non-overlapping CSMs tend to have lower scores on average. Additionally, we accessed the original spectra of these two sets of CSMs and computed their MS2 ion intensities. This analysis demonstrated that non-overlapping CSMs typically have lower ion intensities in the MS2 than their overlapping counterparts (Fig.S3(b)). This suggested that the protein feedback (PF) mechanism in ECL 3.0 enables the identification of these less-well-fragmented spectra and helps discern ambiguities using PF information. As a result, these spectra are the primary beneficiaries of ECL 3.0, specifically the protein feedback mechanism.

Table S1: Parameters used in the analysis of non-cleavable data set (PXD014337).

PXD014337	ECL 3.0	Kojak	pLink 2
Enzyme	Trypsin	Trypsin	Trypsin
Miss_cleavages	2	2	2
Min_length	5	5	5
Fixed Modifications	C+57.02 Da	C+57.02 Da	C+57.02 Da
Variable Modifications	M+15.99 Da	M+15.99 Da	M+15.99 Da
MS1 tolerance	10ppm	10ppm	10ppm
MS2 tolerance	0.02 Da	0.02 Da	0.02 Da
Linker info	DSS $m_{xl} = 138.068$		
Link site	K	K	K
FDR setting	1%	1%	1%

Table S2: Parameters used in the analysis of E. coli data sets.

PXD012109	ECL 3.0	Kojak	pLink 2
Enzyme	Trypsin	Trypsin	Trypsin
Miss_cleavages	2	2	2
Min_length	6	6	6
Fixed Modifications	C+57.02 Da	C+57.02 Da	C+57.02 Da
Variable Modifications	M+15.99 Da	M+15.99 Da	M+15.99 Da
MS1 tolerance	10ppm	10ppm	10ppm
MS2 tolerance	0.02 Da	0.02 Da	0.02 Da
Linker info	Leiker $m_{xl} = 316.142$		
Link site	K	K	K
FDR setting	1%	1%	1%

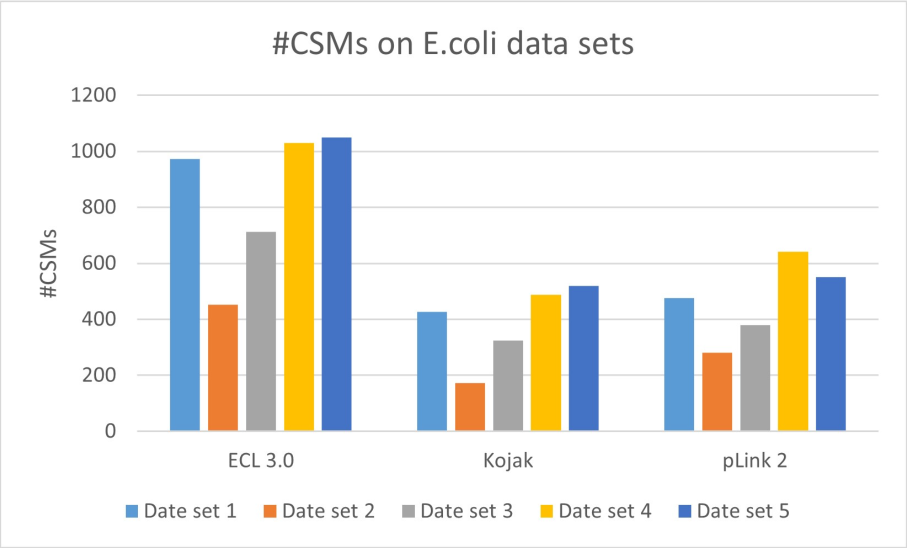

Figure S1: ECL 3.0 is compared with Kojak and pLink 2 using five E. coli data sets. The number of CSMs is calculated for each result. ECL 3.0 outperforms the other two tools.

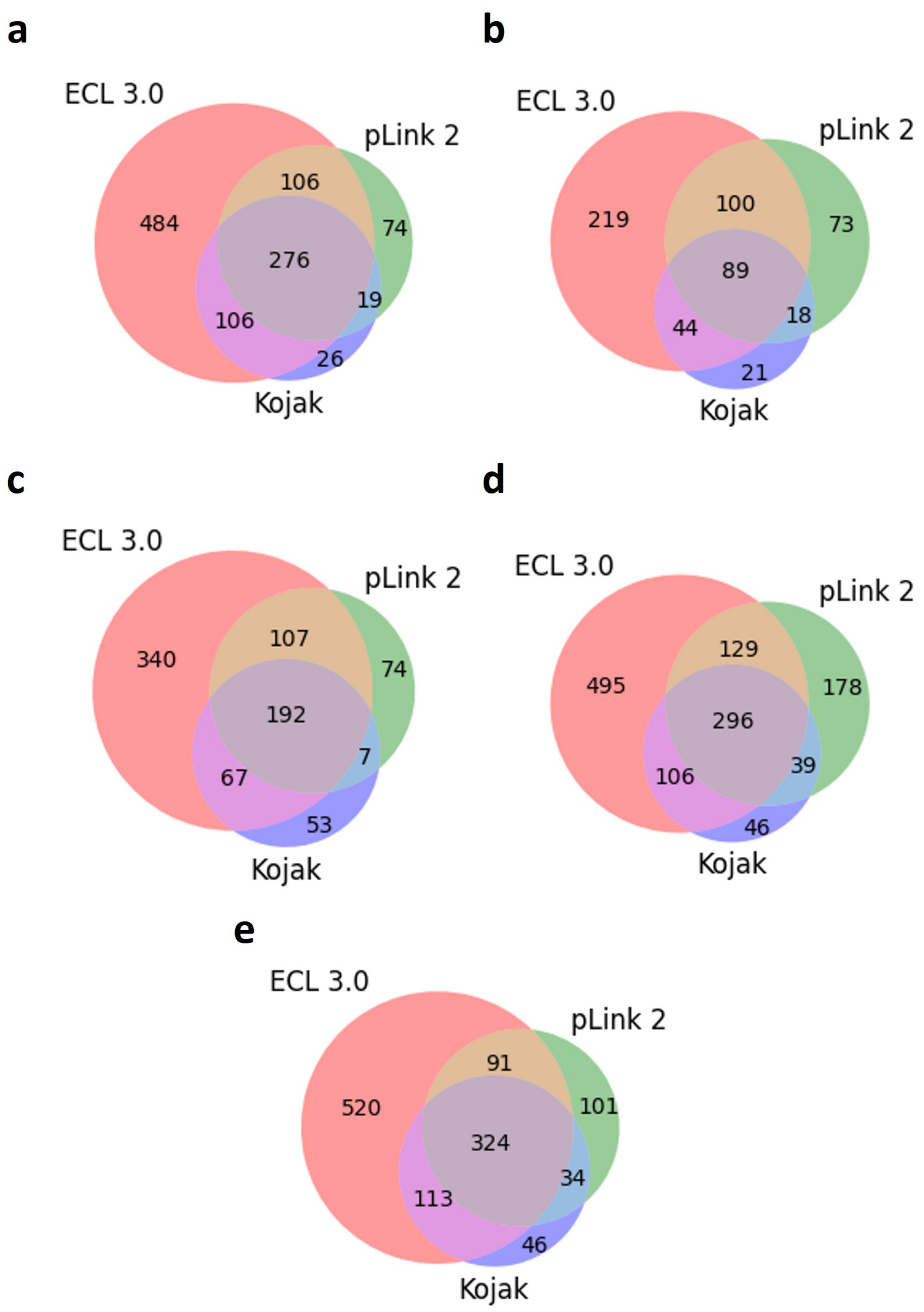

Figure S2: Venn diagrams of three software tools using the E. coli data sets. (a)-(e) Five Venn diagrams of the CSMs results using ECL 3.0, Kojak, and pLink 2 corresponding to the five data sets in Fig. S1. On average, ECL 3.0 covers 73% results of the other two tools in addition to its own unique results.

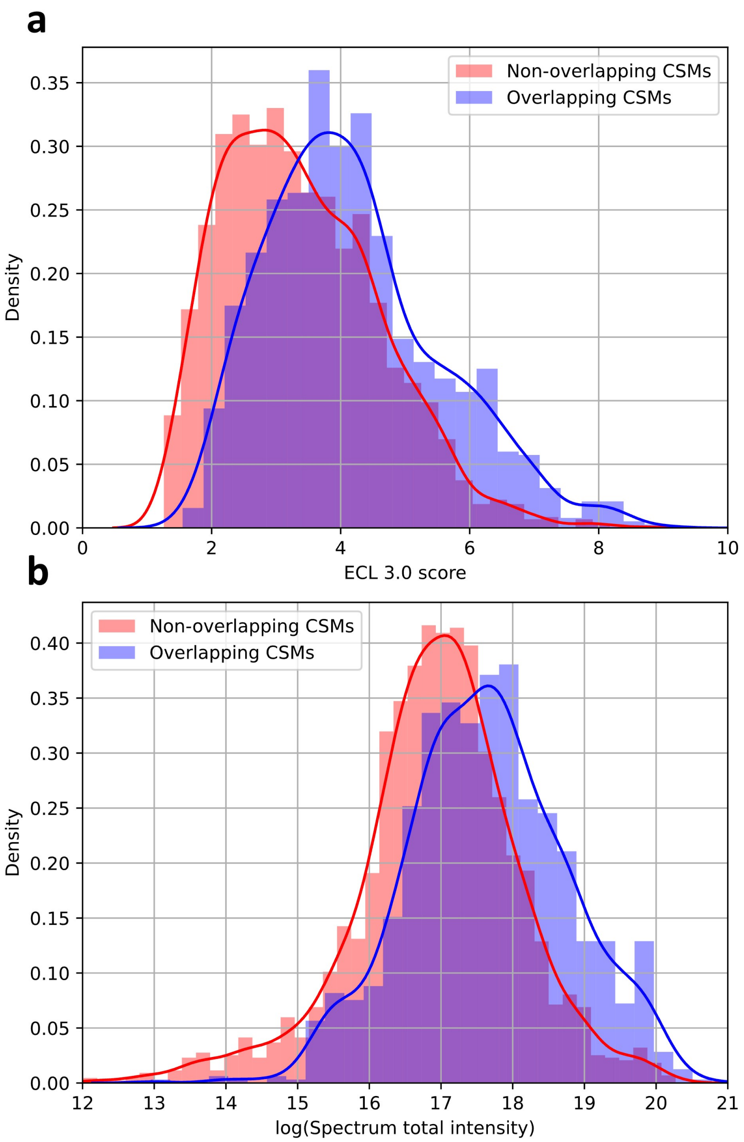

Figure S3: Histograms and distributions of non-overlapping and overlapping CSMs. (a) Score distribution of non-overlapping and overlapping CSMs. It is evident that the non-overlapping CSMs in ECL 3.0 typically have smaller scores compared to overlapping CSMs, indicating that the sequences are less well-matched to the spectra. (b) MS2 ion intensities distribution of non-overlapping and overlapping CSMs. We observe that the ion abundance is frequently lower in non-overlapping spectra compared to overlapping spectra, which partially explains why the scores in non-overlapping CSMs are lower. The density is fitted using kernel density estimation within the seaborn package in the Python language.

Comparison using human data sets

We used three recent large human protein data sets (PXD035433 [4], PXD014675 [5], PXD034327 [6]) to compare the performance among ECL 3.0, Kojak, and pLink 2. The parameters used are shown in Table. S3. The number of CSMs and that of unique cross-linked peptides for each tool are plotted in Fig. S4 and Fig. S5. The results showed that ECL 3.0 identifies the most results using the same quality control setting on these more complicated and larger MS2 spectra.

Table S3: Parameters used in the analysis of human data sets.

	ECL 3.0	Kojak	pLink 2
Enzyme	Trypsin	Trypsin	Trypsin
Miss_cleavages	2	2	2
Min_length	6	6	6
Fixed Modifications	C+57.02 Da	C+57.02 Da	C+57.02 Da
Variable Modifications	M+15.99 Da	M+15.99 Da	M+15.99 Da
MS1 tolerance	10ppm	10ppm	10ppm
MS2 tolerance	0.05 Da	0.05 Da	0.05 Da
Linker info	DSS/BS3 $m_{xl} = 138.068$		
Link site	K	K	K
FDR setting	1%	1%	1%

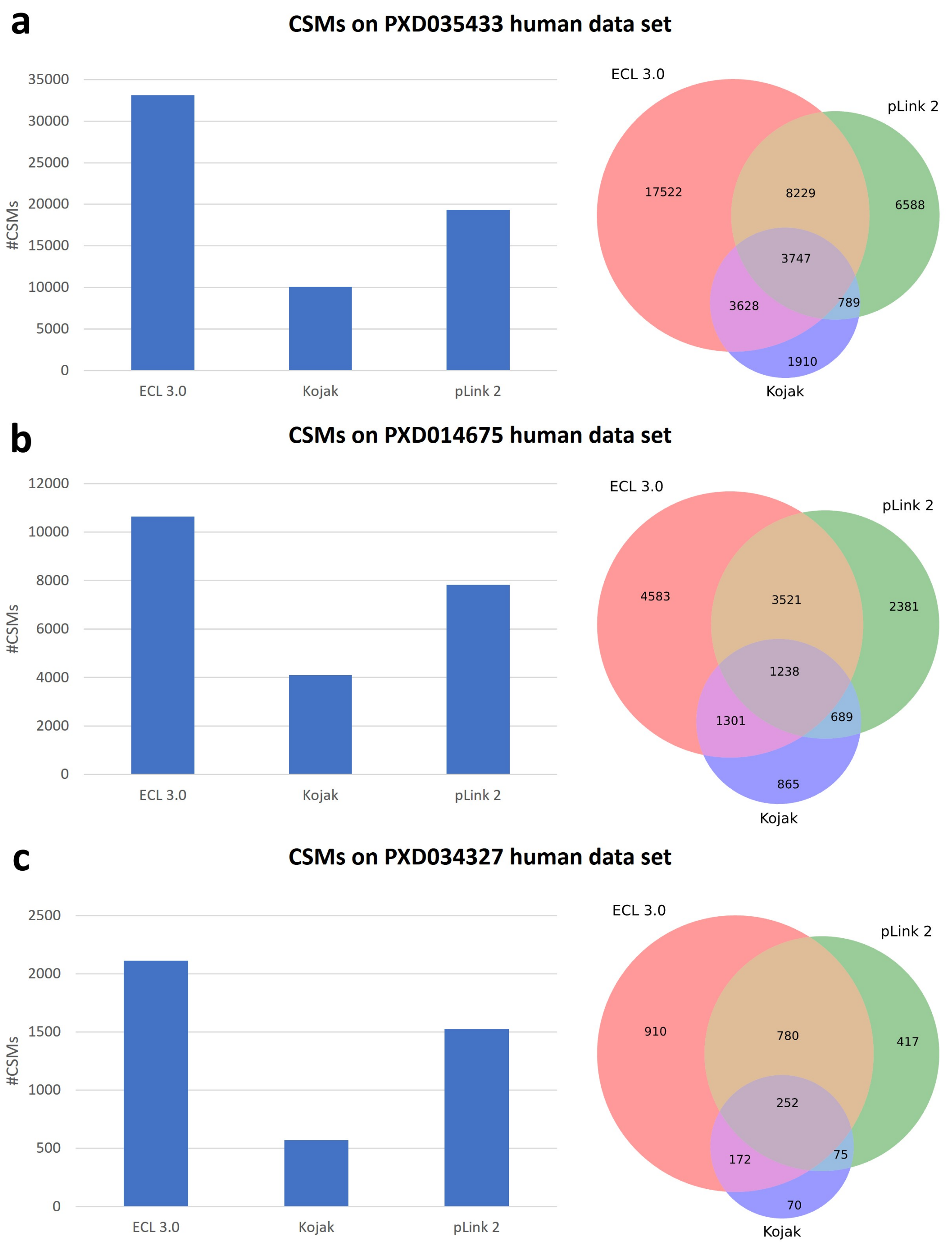

Figure S4: CSMs comparison and Venn diagrams of software tools using three human data sets. (a)-(c) PXD035433, PXD014675 and PXD034327 data sets result are shown individually. ECL 3.0 systematically identified the most CSMs and on average, ECL 3.0 covers 64% results of the other two tools in addition to its own unique results.

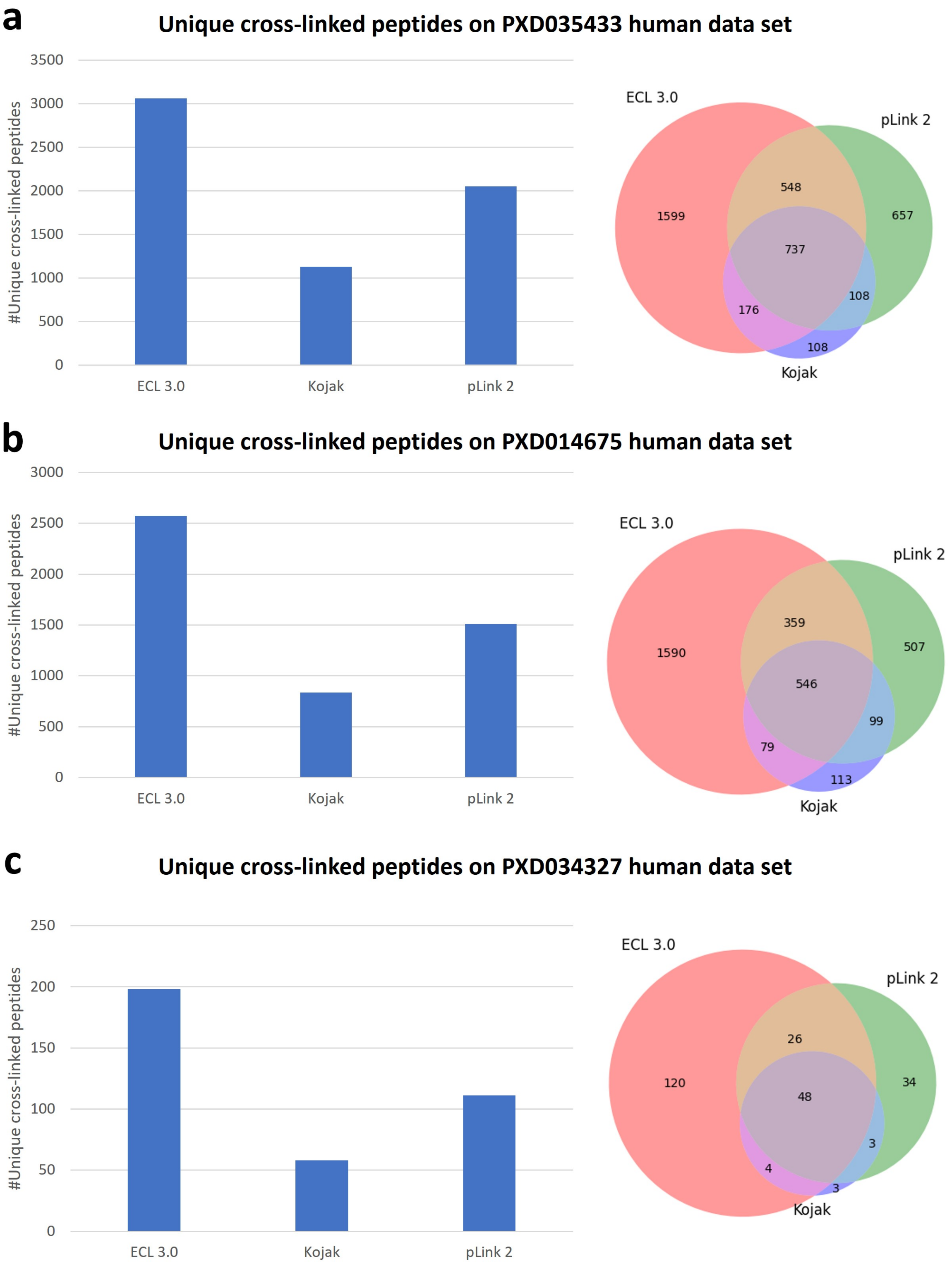

Figure S5: Number of unique cross-linked peptides and Venn diagrams of software tools using three human data sets. (a)-(c) Results using PXD035433, PXD014675 and PXD034327 data sets are shown individually. ECL 3.0 has identified the most number of unique cross-linked peptides.

False positives are under control in the protein feedback method

To validate whether the protein feedback method increases false positives in the CSMs result and affects the distribution of decoy matches, we conducted the following experiment.

First, we combined the E. coli data set which uses the DSSO linker (PXD011861) [7] and the human data set which also uses the DSSO linker (PXD031668) [8] to form a new data set comprising all E. coli MS2 spectra and human MS2 spectra. It contains 128,152 E. coli spectra and 225,419 human spectra, stored in an mzXML file. We analyze the merged data set using ECL 3.0 with the following parameters: MS1 tolerance of 10ppm, MS2 tolerance of 20ppm, 2 missed cleavages, trypsin digestion, fix modification C+57.02Da, variable modification M+15.99Da, and an FDR of 0.01. The database FASTA file encompassed 604 protein sequences downloaded from corresponding proteome repositories, consisting of 171 E. coli proteins and 433 human proteins.

The results revealed that 6,856 MS2 spectra out of 353,571 were successfully identified. Among these, 3,336 CSMs represented human cross-linked peptides, while 3,490 CSMs were attributed to E. coli cross-linked peptides. Additionally, 30 CSMs were identified as mixed cross-linked peptides, indicating a false positive ratio of approximately 0.00437. (human_ecoli_merged.csv)

This experiment showed clear evidence that the protein feedback method does not introduce bias into the distribution of decoy sequences.

References

[1] Zhou, C., Dai, S., Lin, Y., Lian, S., Fan, X., Li, N., Yu, W.: Exhaustive cross-linking search with protein feedback. Journal of Proteome Research **22**, 101–113 (2023)

[2] Chen, Z.-L., Meng, J.-M., Cao, Y., Yin, J.-L., Fang, R.-Q., Fan, S.-B., Liu, C., Zeng, W.-F., Ding, Y.-H., Tan, D., *et al.*: A high-speed search engine plink 2 with systematic evaluation for proteome-scale identification of cross-linked peptides. Nature Communications **10**, 3404–3415 (2019)

[3] Hoopmann, M.R., Zelter, A., Johnson, R.S., Riffle, M., MacCoss, M.J., Davis, T.N., Moritz, R.L.: Kojak: efficient analysis of chemically cross-linked protein complexes. Journal of Proteome Research **14**, 2190–2198 (2015)

[4] Chen, Y., Zhou, W., Xia, Y., Zhang, W., Zhao, Q., Li, X., Gao, H., Liang, Z., Ma, G., Yang, K., *et al.*: Targeted cross-linker delivery for the in situ mapping of protein conformations and interactions in mitochondria. Nature Communications **14**, 3882–3897 (2023)

[5] Ryl, P.S., Bohlke-Schneider, M., Lenz, S., Fischer, L., Budzinski, L., Stuiver, M., Mendes, M.M., Sinn, L., O'reilly, F.J., Rappsilber, J.: In situ structural restraints from cross-linking mass spectrometry in human mitochondria. Journal of Proteome Research **19**, 327–336 (2019)

[6] Chang, Y.-G., Lupton, C.J., Bayly-Jones, C., Keen, A.C., D'Andrea, L., Lucato, C.M., Steele, J.R., Venugopal, H., Schittenhelm, R.B., Whisstock, J.C., *et al.*: Structure of the metastatic factor p-rex1 reveals a two-layered autoinhibitory mechanism. Nature Structural & Molecular Biology **29**, 767–773 (2022)

[7] Stieger, C.E., Doppler, P., Mechtler, K.: Optimized fragmentation improves the identification of peptides cross-linked by ms-cleavable reagents. Journal of Proteome Research **18**, 1363–1370 (2019)

[8] Graziadei, A., Schildhauer, F., Spahn, C., Kraushar, M., Rappsilber, J.: SARS-CoV-2 Nsp1 N-terminal and linker regions as a platform for host translational shutoff. bioRxiv (2022)
